# Supplementary material for: Effects of Electrochemotherapy on Immunologically Important Modifications in Tumor Cells
Source: Vaccines (Basel). 2023 Apr 30;11(5):925. doi: 10.3390/vaccines11050925 (PMC10223734; doi:10.3390/vaccines11050925)
Supplement: Supplementary file 1 [file vaccines-11-00925-s001.zip › vaccines-2348782-supplementary.pdf]

SUPPLEMENTARY INFORMATION

Effects of electrochemotherapy on immunologically important modifications in tumor cells

Ursa Kesar, Bostjan Markelc, Tanja Jesenko, Katja Ursic Valentinuzzi, Maja Cemazar, Primoz Strojan and Gregor Sersa

Supplementary materials

Table S1: Cell densities for clonogenic assay with CDDP and OXA.

		CDDP and OXA									
**Chemotherapeutic concentration (µM)**		**0**	**2.5**	**5**	**10**	**15**	**20**	**30**	**40**	**60**	**80**
No. of cells/well	without EP	75	75	75	75	75	75	100 (75 for B16-F10 with OXA)	150 (75 for B16-F10 with OXA)	75	75
	with EP	100	100	150	150	200	200	300	400	400	400

Table S2: Cell densities for clonogenic assay with BLM.

		BLM						
**Chemotherapeutic concentration (nM)**		**0**	**0.01**	**0.1**	**0.5**	**1.0**	**1.3**	**1.8**
No. of cells/well	without EP	50	75	75	75	75	100	100
	with EP	100	100	150	150	150	200	250

Table S3: List of antibodies used.

Antibodies	Sources	Cat#	Volume per sample (μL)
MHC class I (H-2Kb) Monoclonal Antibody (AF6-88.5.5.3), APC	Thermo Fisher Scientific	17-5958-82	0.5
MHC class I (H-2Kd) Monoclonal Antibody (SF1-1.1.1), APC	Thermo Fisher Scientific	17-5957-82	0.5
MHC class II (I-A/I-E) Monoclonal Antibody (M5/114.15.2), Super Bright 780	Thermo Fisher Scientific	78-5321-82	0.5
Anti-Mouse CD274 (B7-H1), PE 100 μg (PD-L1)	Thermo Fisher Scientific	12-5982-82	0.5
Calreticulin Antibody (DyLight 405), 0.1 mL	Novus Biologicals	NV-NB600-101V	1
CD40 Monoclonal Antibody (1C10), Super Bright 600	Thermo Fisher Scientific	63-0401-82	1.5

Table S4: Determined IC values for all chemotherapeutics.

the

A

CDDP (µM)	IC30	IC50	IC70
**B16-F10**	6.86 ± 0.73	12.90 ± 1.46	17.87 ± 1.93
**4T1**	5.23 ± 1.65	13.80 ± 1.91	22.24 ± 2.67
**CT26**	4.54 ± 0.60	11.70 ± 0.71	17.69 ± 0.78

B

OXA (µM)	IC30	IC50	IC70
**B16-F10**	8.89 ± 2.42	24.46 ± 2.57	39.29 ± 3.58
**4T1**	5.65 ± 0.76	13.95 ± 1.45	18.92 ± 1.10
**CT26**	2.68 ± 0.37	14.62 ± 1.38	23.58 ± 2.07

C

BLM (nM)	IC30	IC50	IC70
**B16-F10**	0.15 ± 0.05	0.34 ± 0.05	0.63 ± 0.04
**4T1**	0.16 ± 0.04	0.35 ± 0.06	0.50 ± 0.10
**CT26**	0.06 ± 0.02	0.27 ± 0.05	0.56 ± 0.07

Supplementary figures

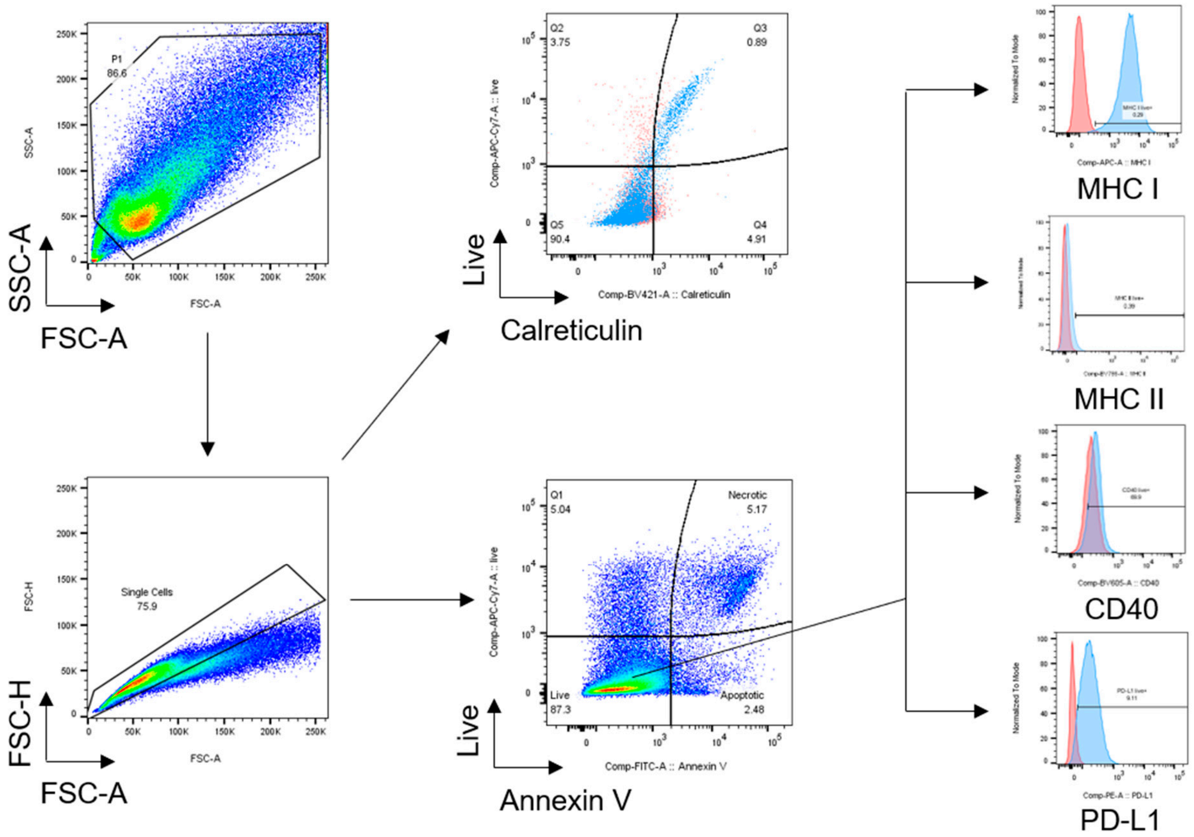

Figure S1: Gating strategy. After ECT experiments were performed, single cells were isolated from debris (SSC-A vs FSC-A) and doublets (FSC-H vs FSC-A). After gating of live and dead cells (efluor 780; APC-Cy7-A vs Annexin V), cells that express MHC I, MHC II, CD40 and PD-L1 were identified. After gating of single cells, cells that express Calreticulin (efluor 780; APC-Cy7-A vs BV421-A) were identified. Percentages shown are derived from the proportion of cells positive for each marker. A total of 100,000 events were collected. SSC-A, side-scatter area; FSC-A, forward-scatter area; FSC-H, forward-scatter height.

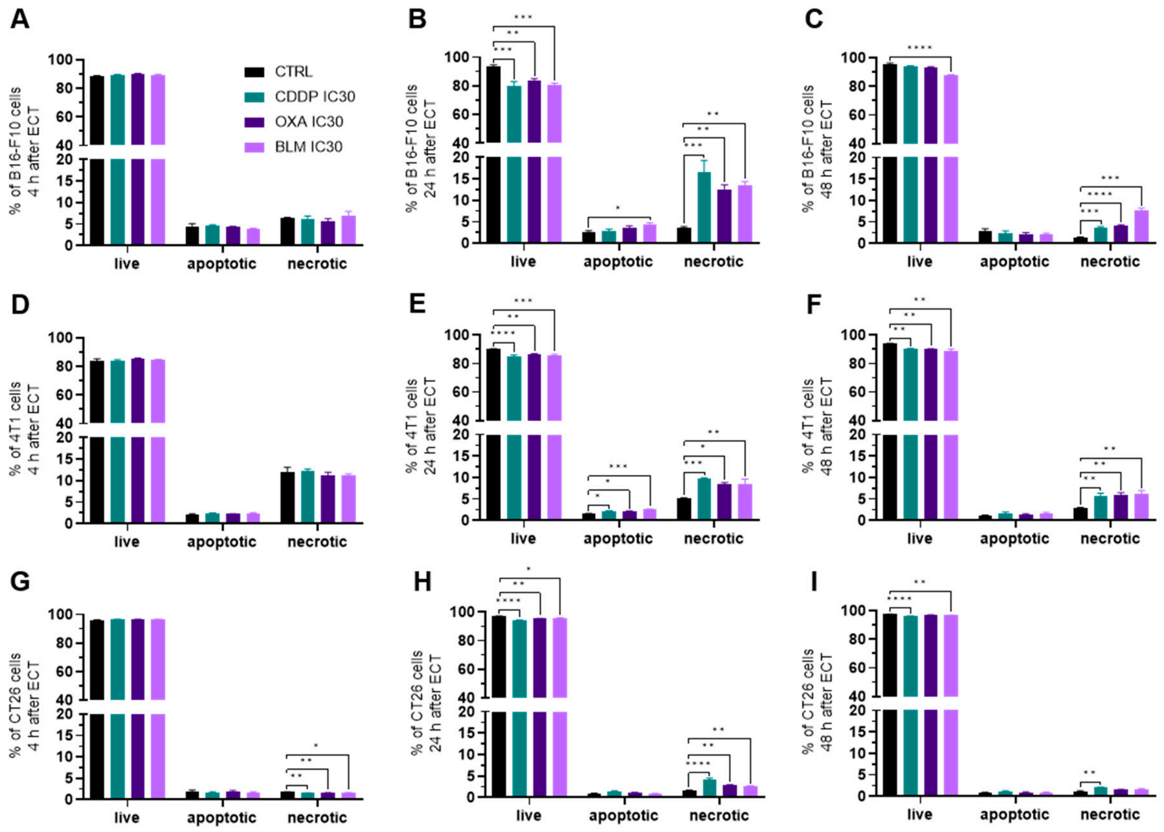

Figure S2: Cell viability at IC_{30} concentrations. Cell viability in B16-F10 cell line 4 h (A), 24 h (B), 48 h (C) after ECT. Cell viability in 4T1 cell line 4 h (D), 24 h (E) and 48 h (F) after ECT. Cell viability in CT26 cell line 4 h (G), 24 h (H) and 48 h (I) after ECT. The values are presented as the AM ± SEM. *$p \leq 0.05$, **$p \leq 0.01$, ***$p \leq 0.001$, ****$p \leq 0.0001$, $n = 4$.

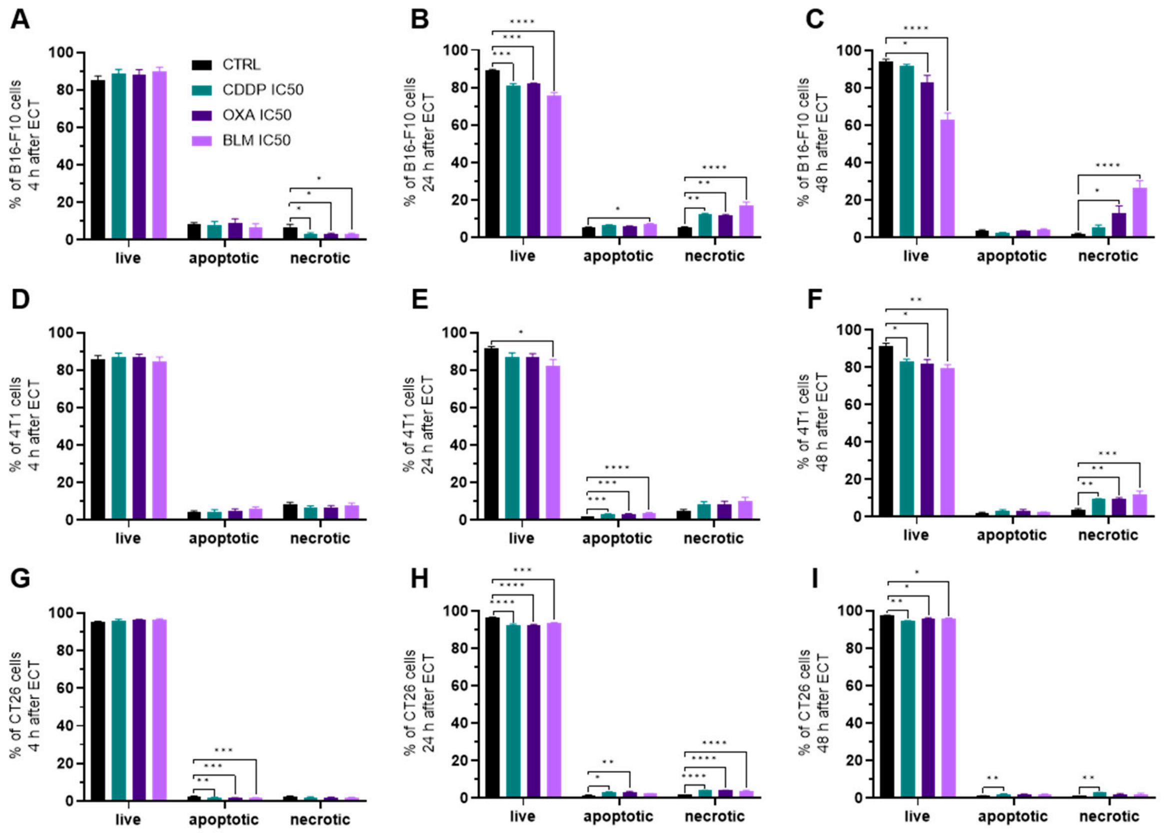

Figure S3: Cell viability at IC$_{50}$ concentrations. Cell viability in B16-F10 cell line 4 h (A), 24 h (B), 48 h (C) after ECT. Cell viability in 4T1 cell line 4 h (D), 24 h (E) and 48 h (F) after ECT. Cell viability in CT26 cell line 4 h (G), 24 h (H) and 48 h (I) after ECT. The values are presented as the AM ± SEM. *$p \leq 0.05$, **$p \leq 0.01$, ***$p \leq 0.001$, ****$p \leq 0.0001$, $n = 4$.

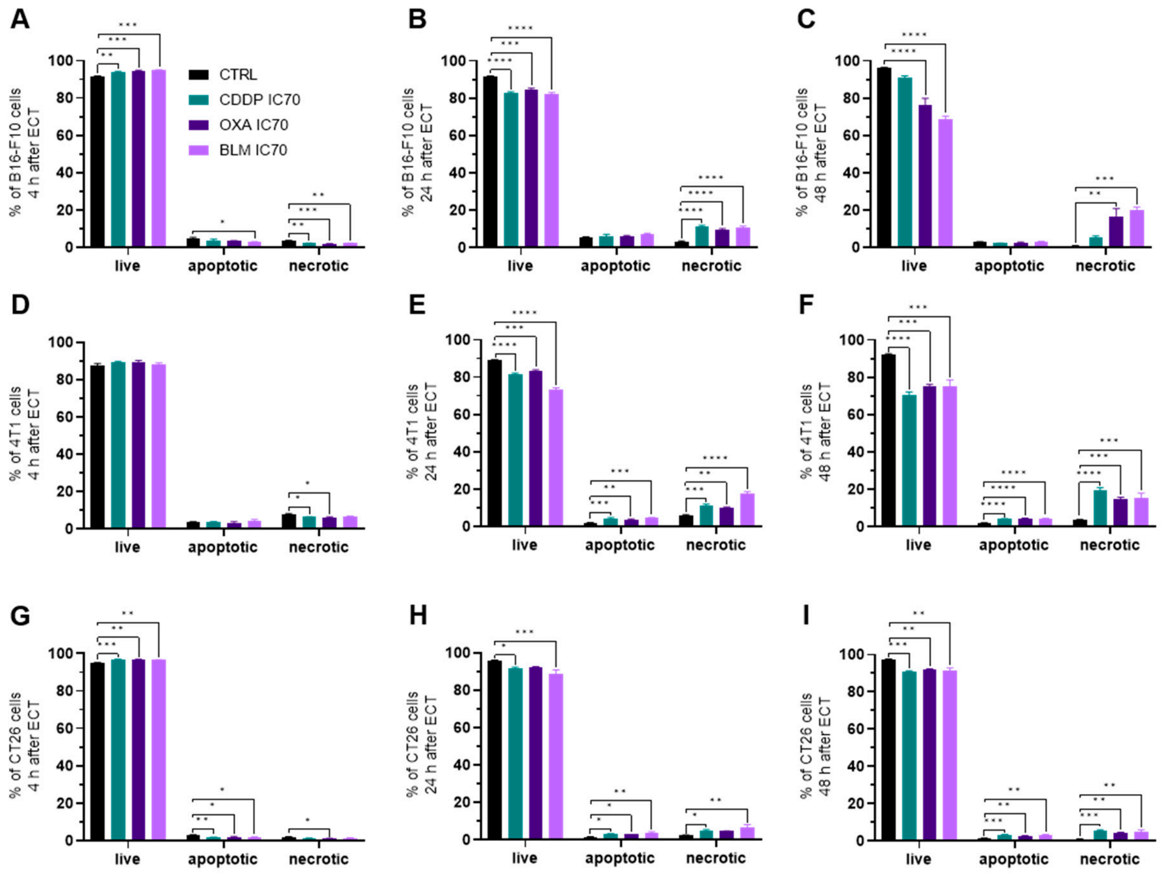

Figure S4: Cell viability at IC_{70} concentrations. Cell viability in B16-F10 cell line 4 h (A), 24 h (B), 48 h (C) after ECT. Cell viability in 4T1 cell line 4 h (D), 24 h (E) and 48 h (F) after ECT. Cell viability in CT26 cell line 4 h (G), 24 h (H) and 48 h (I) after ECT. The values are presented as the AM ± SEM. *$p \leq 0.05$, **$p \leq 0.01$, ***$p \leq 0.001$, ****$p \leq 0.0001$, $n = 4$.

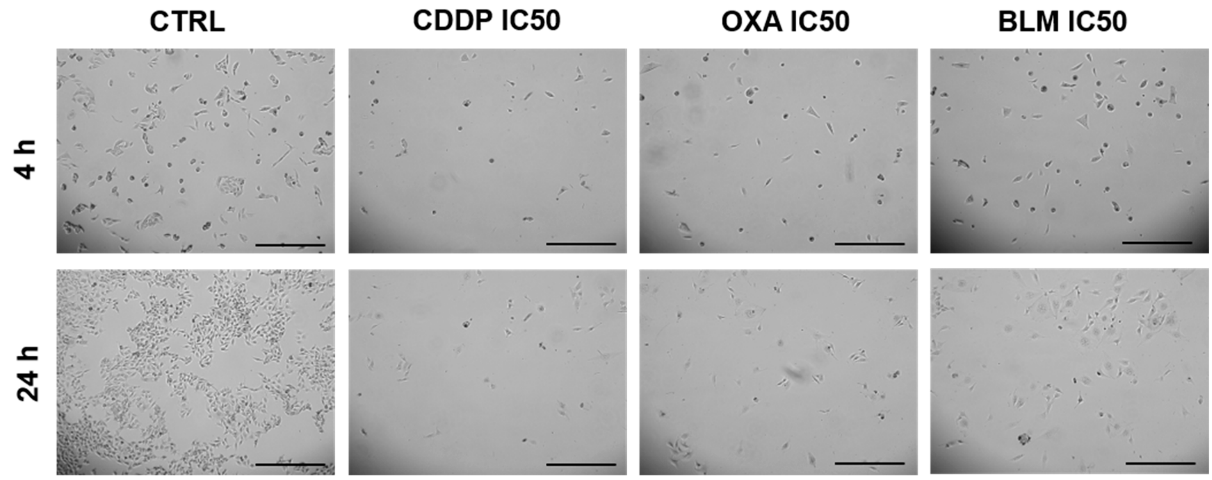

Figure S5: Cell number changes after ECT in the B16-F10 cell line. Cells 4 or 24 h after ECT with IC_{50} concentrations of CDDP, OXA or BLM in comparison with untreated control cells. Scale bar: 0.5 mm.

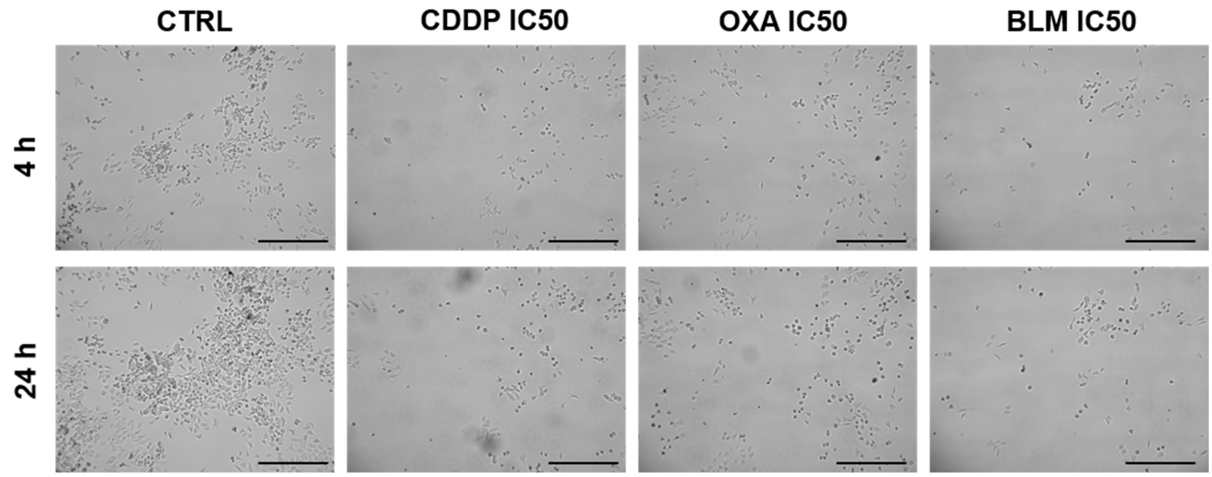

Figure S6: Cell number changes after ECT in the 4T1 cell line. Cells 4 or 24 h after ECT with IC_{50} concentrations of CDDP, OXA or BLM in comparison with untreated control cells. Scale bar: 0.5 mm.

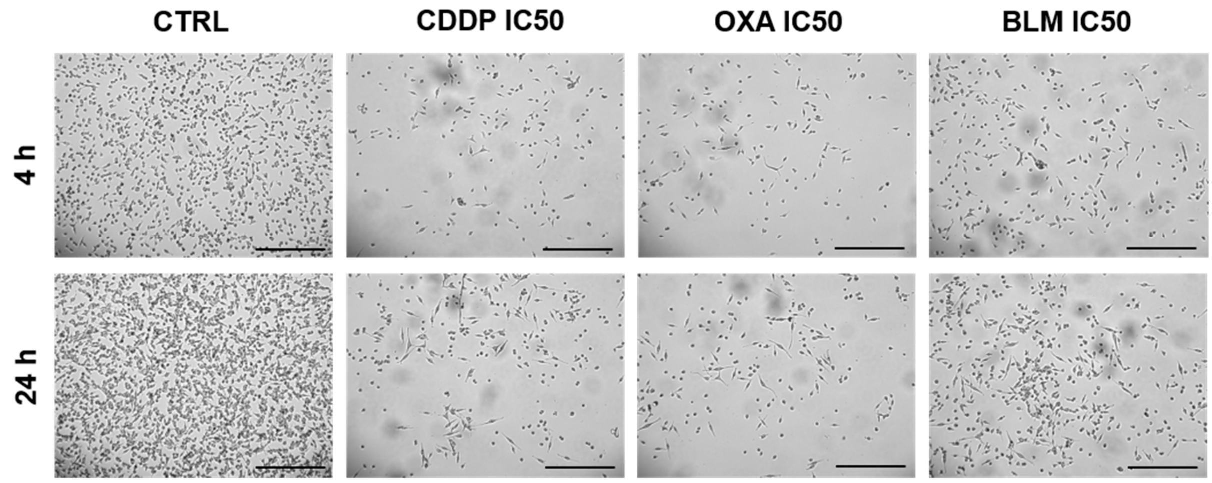

Figure S7: Cell number changes after ECT in the CT26 cell line. Cells 4 or 24 h after ECT with IC_{50} concentrations of CDDP, OXA or BLM in comparison with untreated control cells. Scale bar: 0.5 mm.

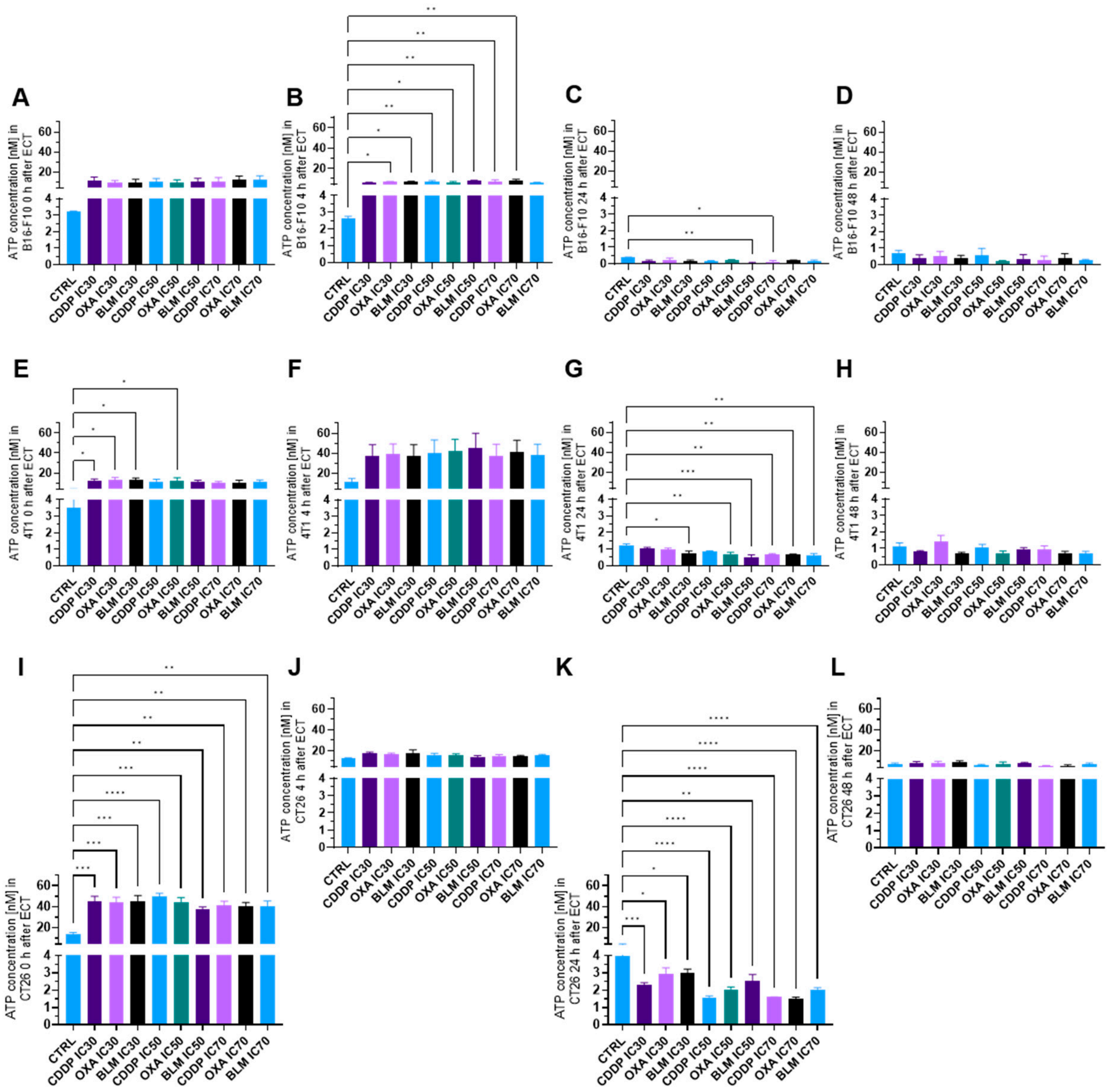

Figure S8: Released ATP concentration in B16-F10, 4T1 and CT26 cell lines. Released ATP concentration in B16-F10 cell line 0 h (A), 4 h (B), 24 h (C) and 48 h (D) after ECT. Released ATP concentration in 4T1 cell line 0 h (E), 4 h (F), 24 h (G) and 48 h (H) after ECT. Released ATP concentration in CT26 cell line 0 h (I), 4 h (J), 24 h (K) and 48 h (L) after ECT. The values are presented as the AM ± SEM. *$p \leq 0.05$, **$p \leq 0.01$, ***$p \leq 0.001$, ****$p \leq 0.0001$, $n = 3$.

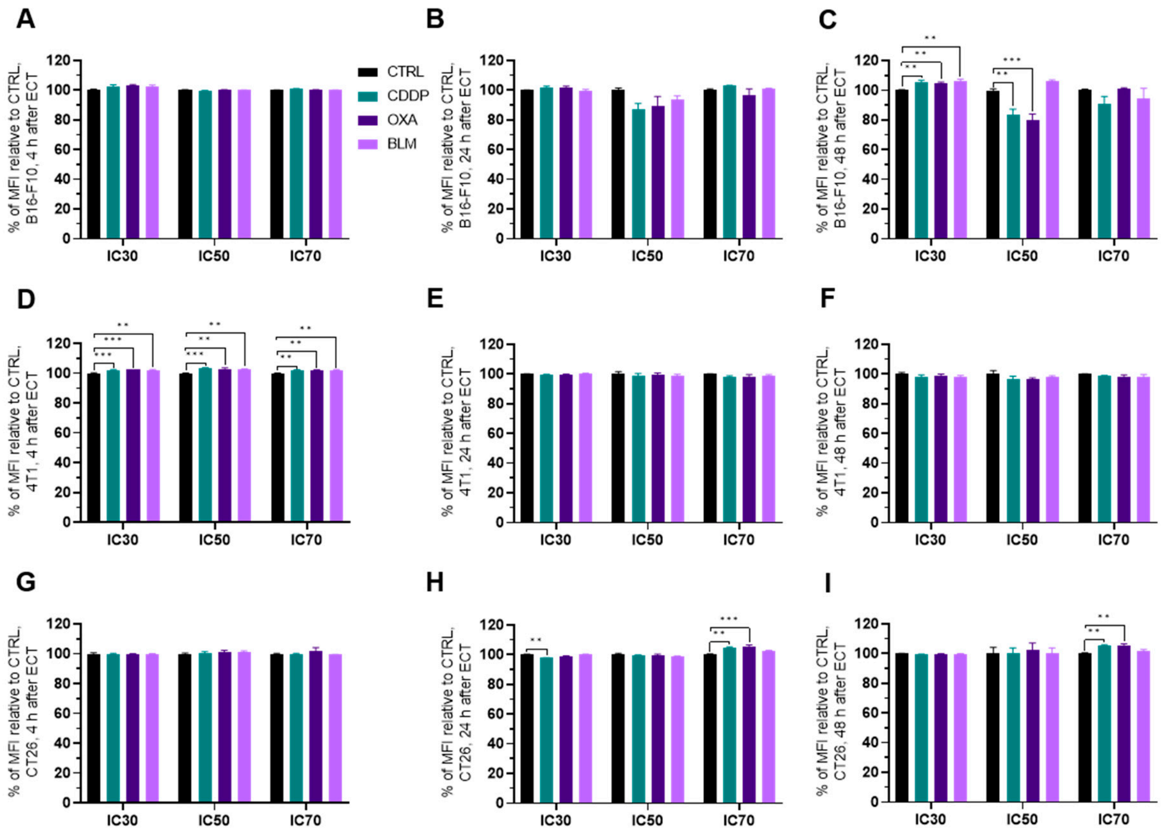

Figure S9: CRT expression after ECT. Calreticulin expression in B16-F10 cell line 4 h (A), 24 h (B), 48 h (C) after ECT. Calreticulin expression in 4T1 cell line 4 h (D), 24 h (E) and 48 h (F) after ECT. Calreticulin expression in CT26 cell line 4 h (G), 24 h (H) and 48 h (I) after ECT. The values are presented as the AM ± SEM. *$p \leq 0.05$, **$p \leq 0.01$, ***$p \leq 0.001$, ****$p \leq 0.0001$, $n = 4$.

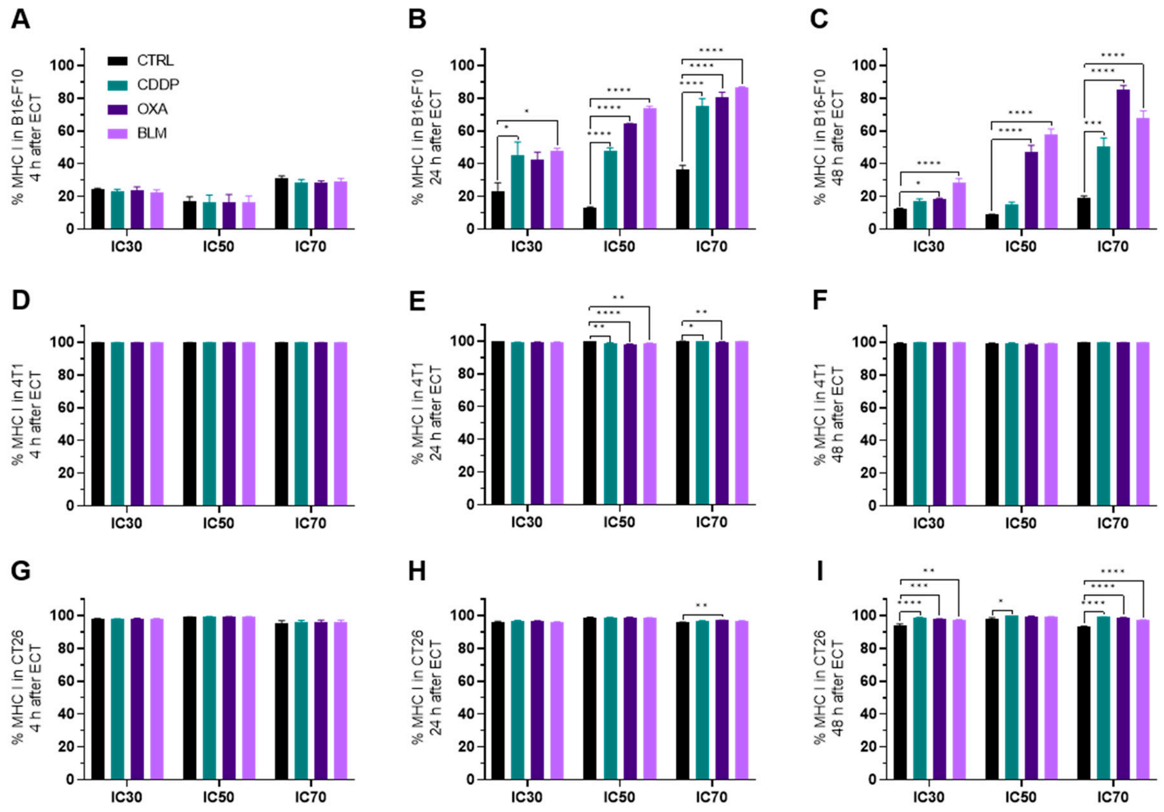

Figure S10: MHC I expression after ECT. Percent of MHC I positive cells is shown. MHC I expression in B16-F10 cell line 4 h (A), 24 h (B), 48 h (C) after ECT. MHC I expression in 4T1 cell line 4 h (D), 24 h (E) and 48 h (F) after ECT. MHC I expression in CT26 cell line 4 h (G), 24 h (H) and 48 h (I) after ECT. The values are presented as the AM ± SEM. *$p \leq 0.05$, **$p \leq 0.01$, ***$p \leq 0.001$, ****$p \leq 0.0001$, $n = 4$.

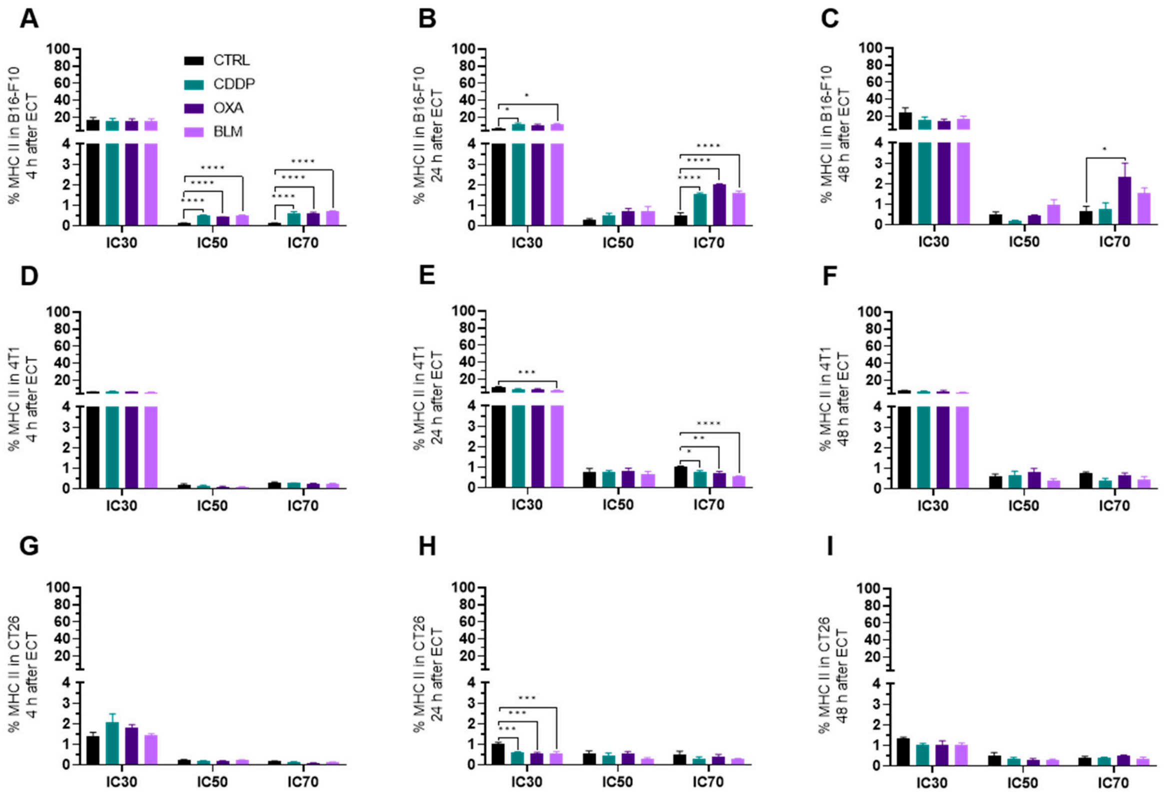

Figure S11: MHC II expression after ECT. Percent of MHC II positive cells is shown. MHC II expression in B16-F10 cell line 4 h (A), 24 h (B), 48 h (C) after ECT. MHC II expression in 4T1 cell line 4 h (D), 24 h (E) and 48 h (F) after ECT. MHC II expression in CT26 cell line 4 h (G), 24 h (H) and 48 h (I) after ECT. The values are presented as the AM ± SEM. *p ≤ 0.05, **p ≤ 0.01, ***p ≤ 0.001, ****p ≤ 0.0001, $n = 4$.

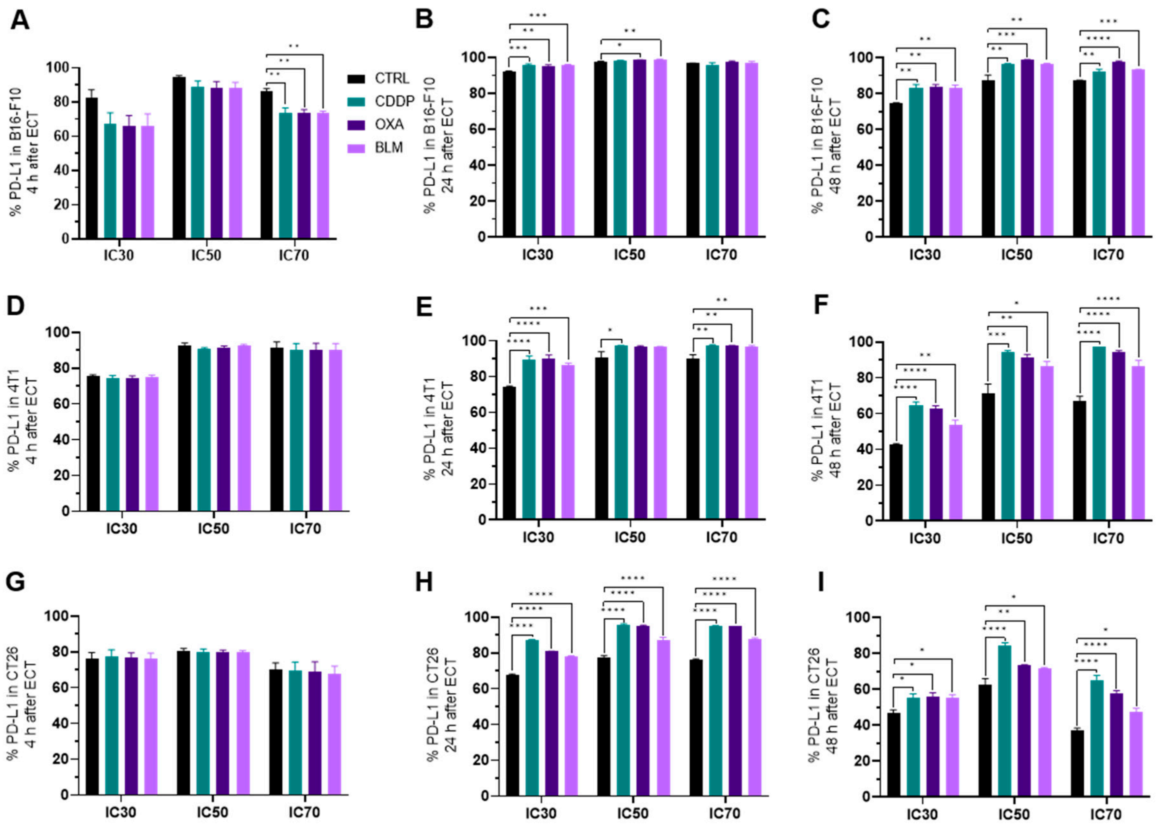

Figure S12: PD-L1 expression after ECT. PD-L1 expression in B16-F10 cell line 4 h (A), 24 h (B), 48 h (C) after ECT. PD-L1 expression in 4T1 cell line 4 h (D), 24 h (E) and 48 h (F) after ECT. PD-L1 expression in CT26 cell line 4 h (G), 24 h (H) and 48 h (I) after ECT. The values are presented as the AM ± SEM. $*p \leq 0.05$, $**p \leq 0.01$, $***p \leq 0.001$, $****p \leq 0.0001$, $n = 4$.

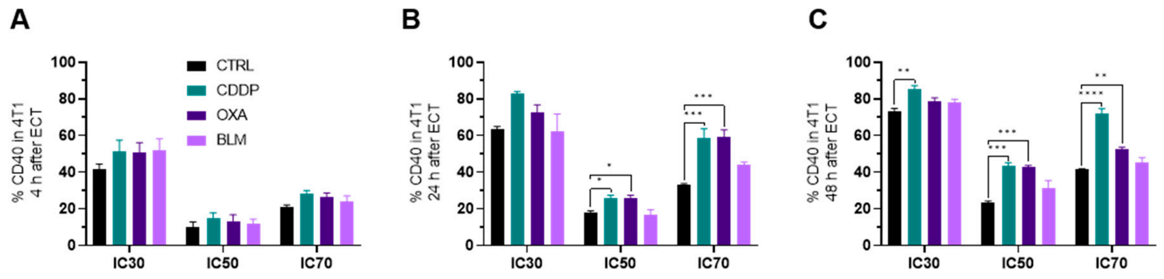

Figure S13: CD40 expression in the 4T1 cell line 4 h (A), 24 h (B), 48 h (C) after ECT. The values are presented as the AM ± SEM. $^{*}p \le 0.05$, $^{**}p \le 0.01$, $^{***}p \le 0.001$, $^{****}p \le 0.0001$, $n = 4$.
